# Evaluation of a novel intramuscular prime/intranasal boost vaccination strategy against influenza in the pig model

**DOI:** 10.1371/journal.ppat.1012393

**Published:** 2024-08-08

**Authors:** Robin Avanthay, Obdulio Garcia-Nicolas, Nicolas Ruggli, Llorenç Grau-Roma, Ester Párraga-Ros, Artur Summerfield, Gert Zimmer

**Affiliations:** 1 Institute of Virology and Immunology IVI, Mittelhäusern, Switzerland; 2 Department of Infectious Diseases and Pathobiology, Vetsuisse Faculty, University of Bern, Bern, Switzerland; 3 Graduate School for Cellular and Biomedical Sciences, University of Bern, Bern, Switzerland; 4 Institute of Animal Pathology, COMPATH, Vetsuisse Faculty, University of Bern, Bern, Switzerland; 5 Department of Anatomy and Comparative Pathology, Veterinary Faculty, University of Murcia, Murcia, Spain; Icahn School of Medicine at Mount Sinai, UNITED STATES OF AMERICA

## Abstract

Live-attenuated influenza vaccines (LAIV) offer advantages over the commonly used inactivated split influenza vaccines. However, finding the optimal balance between sufficient attenuation and immunogenicity has remained a challenge. We recently developed an alternative LAIV based on the 2009 pandemic H1N1 virus with a truncated NS1 protein and lacking PA-X protein expression (NS1(1–126)-ΔPAX). This virus showed a blunted replication and elicited a strong innate immune response. In the present study, we evaluated the efficacy of this vaccine candidate in the porcine animal model as a pertinent *in vivo* system. Immunization of pigs via the nasal route with the novel NS1(1–126)-ΔPAX LAIV did not cause disease and elicited a strong mucosal immune response that completely blocked replication of the homologous challenge virus in the respiratory tract. However, we observed prolonged shedding of our vaccine candidate from the upper respiratory tract. To improve LAIV safety, we developed a novel prime/boost vaccination strategy combining primary intramuscular immunization with a haemagglutinin-encoding propagation-defective vesicular stomatitis virus (VSV) replicon, followed by a secondary immunization with the NS1(1–126)-ΔPAX LAIV via the nasal route. This two-step immunization procedure significantly reduced LAIV shedding, increased the production of specific serum IgG, neutralizing antibodies, and Th1 memory cells, and resulted in sterilizing immunity against homologous virus challenge. In conclusion, our novel intramuscular prime/intranasal boost regimen interferes with virus shedding and transmission, a feature that will help combat influenza epidemics and pandemics.

## Author summary

Nasally administered live-attenuated influenza vaccines induce a mucosal immune response that can effectively prevent primary infection and virus shedding. However, the development of alternative live vaccine strategies must consider the balance between eliciting a robust immune response and reducing the virulence of the vaccine candidate. To address this problem, we have developed a novel prime/boost vaccination strategy consisting of an initial intramuscular immunization with a propagation-defective RNA virus vector and subsequent nasal immunization with a modified attenuated influenza virus that has lost its ability to counteract the host innate immune response. Using the porcine model, we demonstrate that this novel strategy is capable of eliciting a robust immune response both systemically and at mucosal surfaces. Importantly, intranasal infection with homologous challenge virus remained undetectable in vaccinated animals. In conclusion, our innovative vaccination regimen represents a promising strategy to control influenza disease and virus spread in both humans and livestock.

## Introduction

Influenza A virus (IAV) causes acute respiratory infections in humans, usually associated with sudden high fever, muscle pain, headache, coughing, and fatigue [1,2]. These symptoms begin one to four days after exposure to the virus and can last for about 2 to 8 days. However, IAV can cause life-threatening infections, in particular if the virus spreads to the lower respiratory tract, which can lead to viral pneumonia and acute respiratory distress syndrome (ARDS) [3–5]. Persons at high risk of severe influenza include the very young (< 2 years of age), the elderly (>65 years of age) or persons with underlying diseases such as diabetes, asthma, and cardiovascular disease. IAV infections occur as seasonal epidemics beginning in November and lasting until March in the northern hemisphere. The cold season is associated with staying indoors, breathing dry air and having closer contacts, all factors that favor aerosol transmission and increase infection rates [6,7].

The most commonly used human influenza vaccines to control seasonal epidemics are inactivated influenza vaccines standardized for hemagglutinin (HA) content [8,9]. These vaccines are administered to individuals before the influenza season by a single intramuscular injection, usually without adjuvant. Immunization triggers the production of serum antibodies that predominantly bind to the HA globular head domain and have virus-neutralizing activity [10,11]. A small proportion of these virus-specific serum IgG is secreted into the lower respiratory tract where they protect against severe influenza pneumonia and ARDS [12–15]. However, secretion of serum IgG into the mucosal tissues of the upper respiratory tract is not efficient [16–18]. In particular, several weeks after immunization, as IgG titers decline, the concentration of secreted IgG is usually not high enough to neutralize virus in the mucosal tissues [19]. As a result, infectious virus may be shed from the upper respiratory tract, leading to potential transmission to other people. Not only are these inactivated vaccines unable to interrupt the chain of infection during seasonal epidemics, they can also drive antigenic drift by selecting for viral escape mutants that harbor typical mutations in the HA globular head domain [20–22]. To compete with the constant antigenic drift of HA, influenza vaccines need to be updated every year. However, as the seed viruses are usually selected six months in advance, there is a significant risk of antigenic mismatch with circulating IAV strains [23,24]. In some seasons, the effectiveness of influenza vaccines may be relatively low, especially in the elderly population [25,26].

Live-attenuated influenza vaccines (LAIV) offer several advantages over inactivated influenza vaccines because they are administered via the natural route of infection and induce a local immune response directed against multiple viral antigens [27–29]. HA-specific IgA secreted into the mucosal tissues of the respiratory tract can neutralize the virus at the site of entry and prevent viral shedding and dissemination [29]. LAIV-induced resident T cell responses directed against conserved influenza antigens can provide some protection against antigen-drifted and heterosubtypic IAV [30,31]. In addition, immunization with LAIV has been shown to improve duration of immunity due to prolonged antigenic stimulation and improved T cell help required to generate long-lived plasma cells [32,33]. Finally, LAIVs do not require adjuvants that can cause local and systemic side effects [34]. However, these benefits require LAIVs to have adequate replicative capacity. Over-attenuation due to limited replication of the vaccine virus will reduce viral antigen production to levels too low to elicit an effective immune response. On the other hand, insufficiently attenuated vaccine candidates could be shed from the upper respiratory tract and transmitted to individuals with a compromised immune system [35]. Uncontrolled replication may also be problematic in terms of the emergence of revertant viruses that have regained virulence or may increase the risk of reassortment events in the case of co-infection with circulating seasonal viruses. Thus, finding an appropriate balance between sufficient immunogenicity and avoiding safety hazards remains a challenge for LAIV development.

LAIV based on cold-adapted viruses with temperature-sensitive mutations in the RNA polymerase genes have been licensed but are recommended only for individuals between 2 and 49 years of age [36,37]. There is also evidence that pre-existing cross-reactive immunity, present in most adults, interferes with replication of this LAIV, thereby reducing vaccine efficacy [37]. Thus, the elderly population is excluded from vaccination with these LAIVs, even though this population is particularly affected by severe influenza.

Other LAIV strategies have taken advantage of recombinant IAV with a modified non-structural 1 (NS1) protein [38–40]. NS1 is a multifunctional protein and virulence factor that counteracts the innate immune response of the host [41–43]. Infection of cells by IAV with a deletion of the NS1 gene efficiently triggers the synthesis and secretion of type I and/or type III interferon (IFN-I, IFN-III) with the result that viral replication is strongly attenuated in immunocompetent hosts [41,44,45]. This attenuation could compromise the immunogenicity of LAIV candidates with NS1 gene deficiency by severely reducing antigen expression. Therefore, LAIV candidates encoding C-terminally truncated NS1 proteins have been generated as they are less attenuated [40,46]. LAIV vaccines based on A/swine/Texas/4199-2/98 (H3N2) (sTX98) encoding a truncated NS1 protein were shown to protect pigs against challenge with the homologous virus [47,48] and to provide protection against a heterosubtypic H1N2 virus [49,50]. Such a vaccine was licensed for use in piglets in the US in 2017 (Ingelvac Provenza, Boehringer Ingelheim), but was withdrawn from the market following the discovery of reassortant viruses with gene segments derived from the vaccine and field strains [51,52]. A recent study showed that NS1-truncated sTX98 replicated for a similar length of time as wild-type virus [53].

In previous work, we generated the recombinant LAIV candidate NS1(1–126) based on the pandemic A/Hamburg/4/2009 (H1N1) strain (pH1N1/09) encoding a C-terminally truncated NS1 protein, or NS1(1–126) combined with a mutation in the PA gene that prevents PA-X expression, NS1(1–126)-ΔPAX [54]. These recombinant viruses showed attenuated viral replication, reduced apoptotic cell death, but enhanced induction of the innate immune response in a porcine bronchiolar epithelial cell line. Based on these characteristics, we proposed that these viruses could represent perfectly engineered LAIV candidates [54].

The aim of the present study was to evaluate these LAIV candidates *in vivo* using pigs as an animal model. Although both LAIVs were highly immunogenic, they appeared to be insufficiently attenuated with respect to the level and duration of vaccine virus shedding. We therefore developed a novel prime/boost vaccination protocol based on an initial intramuscular immunization with a propagation-defective vesicular stomatitis virus (VSV) vector encoding the pH1N1/09 HA antigen and a boosting intranasal immunization with the LAIV candidates. The selection of the VSV vector was based on our previous work demonstrating an efficient induction of antibody and T cell responses to the HA antigen that mediated partial protection of pigs against heterologous IAV challenge [55]. This novel prime/boost vaccination protocol elicited a strong systemic and mucosal immune response and resulted in sterilizing immunity in the vaccinated host.

## Results

### Respiratory tract shedding of LAIV NS1(1–126)

**Fig 1:**
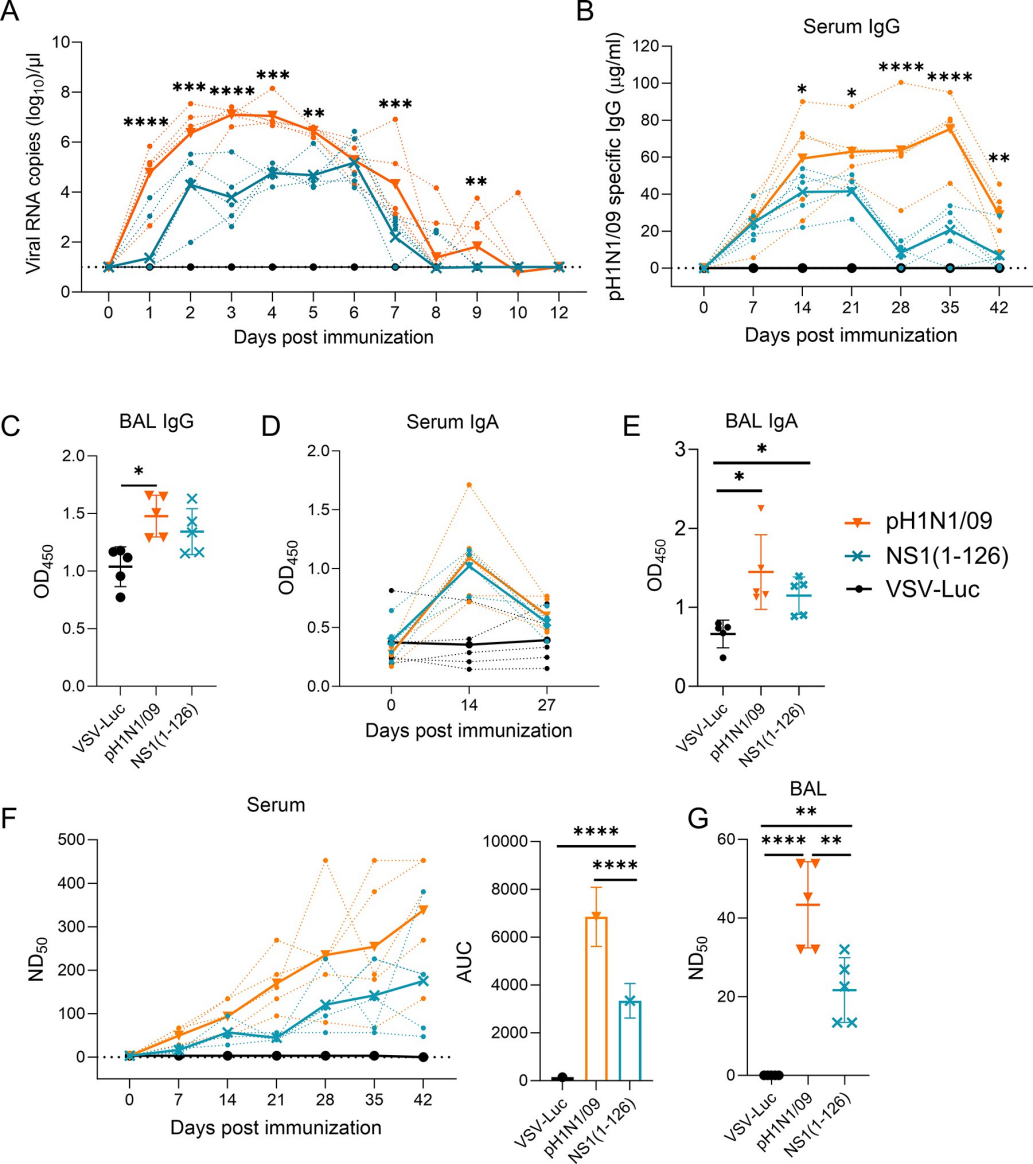
Replication and immunogenicity of NS1(1–126) LAIV in pigs following intranasal administration. The indicated animal groups (group size n = 5) were either immunized via the intramuscular route with VSV-Luc (black symbols) or via the intranasal route with pH1N1/09 (orange symbols) or NS1(1–126) LAIV (turquoise symbols). Nasal swabs samples were collected daily and serum samples at weekly time intervals. At day 42, the animals were euthanized and BAL fluid was collected. (A) Viral RNA quantities in nasal swab samples. The dotted lines show the viral genome copies for the animals at each time point. The bold lines represent the mean values per animal group for each time point. (B, C) Detection of pH1N1/09-specific IgG in sera (B) and BAL fluid (C) by ELISA. The bold lines in (B) represent the mean values for each animal group at each time point. (D, E) Detection by ELISA of pH1N1/09-specific IgA in serum (D) and BAL fluid (E). The dotted lines show the optical density at 450 nm (OD_450_) for all animals at all time points analyzed. The bold lines represent the mean values for each animal group for each time point. (F) Determination of pH1N1/09-neutralizing antibody titres in serum. The neutralizing antibody titres are shown for the individual animal at all time points analyzed (left panel, dotted lines). The mean values are shown as bold lines. The right panel presents the area under the curve (AUC) analysis encompassing all days. (G) Determination of pH1N1/09-neutralizing antibody titres in BAL fluid. Statistical analysis was performed using the two-way ANOVA test (A, B, D) and the one-way ANOVA test (C, E, F right panel, G). *p<0.05, **p<0.01, ***p<0.001, ****p<0.0001 indicate significant differences.

In recent work, we demonstrated that recombinant pH1N1/09 encoding a modified NS1 protein that was truncated by 93 amino acids at the C-terminus replicated in a porcine bronchiolar cell line as efficiently as wild-type virus despite the induction of IFN-I and IFN-III [54]. To determine whether this LAIV candidate would also be attenuated *in vivo*, we infected pigs via the nasal route with either wild-type pH1N1/09 or the NS1(1–126) mutant. Monitoring of rectal temperature for 12 days revealed no fever in any of the animal groups (S1 Fig). Analysis of nasal swab samples by RT-qPCR showed that pH1N1/09 was shed for up to 10 days post infection (Fig 1A and S1 Table). Compared to wild-type virus, the NS1(1–126) mutant was excreted at significantly lower levels with a reduction of viral RNA by almost two log_10_ between days 2 and 5, and a shortening of the overall excretion time by two days. Analysis of serum IgG by ELISA revealed that the NS1(1–126) mutant induced significantly lower levels of pH1N1/09-specific antibodies than wild-type virus (Fig 1B). Over time the serum IgG levels induced by the LAIV candidate dropped faster than those induced by wild-type virus. Nevertheless, in the bronchoalveolar lavage (BAL), the levels of virus-specific IgG were similarly high in both groups (Fig 1C). Likewise, virus-specific serum and BAL IgA levels did not significantly differ between pH1N1/09- and NS1(1–126)-infected animals (Fig 1D and 1E). We also determined the virus-neutralizing antibody titers in serum and BAL fluid and found that animals of the NS1(1–126) group had significantly lower neutralizing antibody levels than animals of the pH1N1/09 group (Fig 1F and 1G). With respect to serum antibodies, the differences between the animal groups were only significant when the area under the curve (AUC) was analyzed (Fig 1F, right panel**)**. Altogether, these data indicate that the LAIV candidate NS1(1–126) was shed from the respiratory tract for considerable time, albeit at reduced levels when compared to wild-type virus. Additionally, the systemic antibody response to the LAIV candidate was reduced compared to the antibody response that was triggered by infection with the wild-type virus.

### Reduction of shedding following intramuscular prime/intranasal boost vaccination

**Fig 2:**
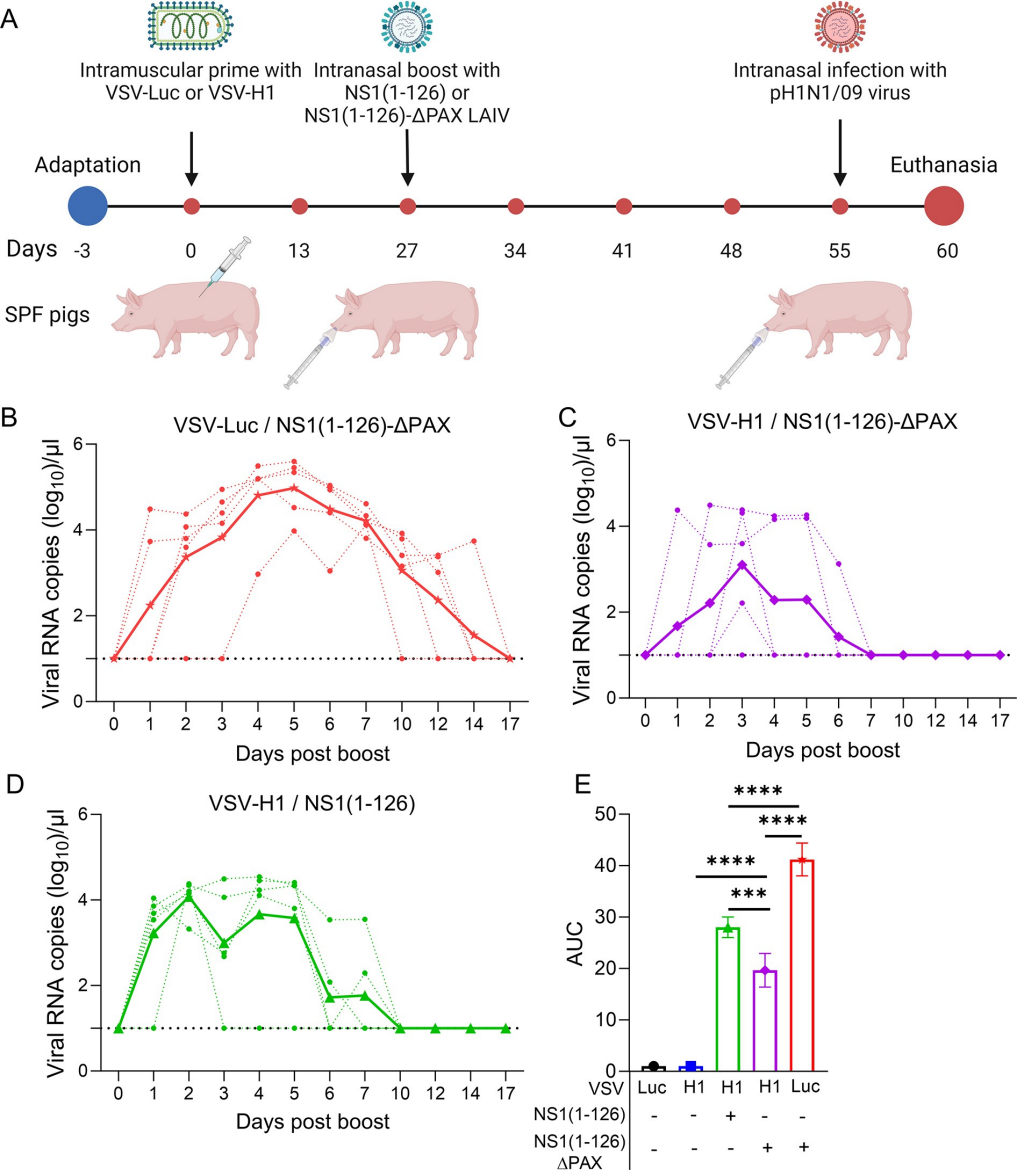
Intramuscular prime/intranasal boost vaccination protocol reduces LAIV shedding. (A) Schematic representation of the experimental design. Red points on the timeline indicate the time points of blood sampling. (B-D) Detection of viral RNA copies in nasal swab samples collected after intranasal inoculation of the animals with the indicated LAIV. At day 55 all animals were challenged via the nasal route using 10^6^ ffu of pH1N1/09. Individual animals are represented by dashed lines and group mean values by continuous thick lines. (B) Pigs were first immunized (i.m.) with the VSV-Luc control vaccine followed by intranasal immunisation with NS1(1–126)-ΔPAX LAIV. (C) Pigs were immunized (i.m.) with VSV-H1 and subsequently boosted (i.n.) with NS1(1–126)-ΔPAX LAIV. (D) Animals were primed (i.m.) with VSV-H1 and boosted (i.n.) with NS1(1–126) LAIV. (E) AUC analyses of viral RNA load in nasal swab samples collected between days 0 and 17 after intranasal vaccination with LAIV (calculated with data from B-D). Significant differences of the AUC values were determined with the one-way ANOVA test (*p<0.05, **p<0.01, ***p<0.001, ****p<0.0001). Fig 2A was created with Biorender.com.

To improve the performance of LAIV in terms of immunogenicity and reduced virus shedding, we developed a novel prime/boost immunization protocol (Fig 2A). The animals were first immunized via the intramuscular route with VSV-H1, a propagation-incompetent VSV vector encoding the pH1N1/09 HA in place of the VSV glycoprotein G. Control animals were immunized with VSV-Luc encoding firefly luciferase. Immunization of pigs with either VSV-H1 or VSV-Luc did not cause any increase of body temperature (S2A Fig) or any other signs of disease. Four weeks after the primary vaccination, the pigs were immunized via the nasal route with the NS1(1–126) or NS1(1–126)-ΔPAX LAIV candidates (Table 1). No fever was recorded for the first 10 days following infection with any of the LAIV (S2B Fig). Detection of viral RNA in nasal swab samples by RT-qPCR showed that animals which were first primed with the control vector VSV-Luc and subsequently immunized via the nasal route with NS1(1–126)-ΔPAX shed virus at relatively high levels and for prolonged time (Fig 2B). In contrast, when the pigs were primed with VSV-H1 and then boosted with NS1(1–126)-ΔPAX, virus shedding was significantly reduced with no viral RNA detectable after day 6 (Fig 2C). Two out of five pigs showed virus shedding for only one day while one pig did not shed any virus for the whole time period surveyed (Fig 2C). Interestingly, if VSV-H1-primed animals were boosted with NS1(1–126) (Fig 2D), they shed higher levels of LAIV than animals that had received the VSV-H1/NS1(1–126)-ΔPAX prime/boost vaccination regimen (Fig 2E). Together, these data demonstrate that shedding of LAIV can be efficiently reduced by our intramuscular prime/intranasal boost vaccination regimen.

**Table 1:** Vaccine used for the prime/boost immunization regimen.

Animal group	Prime vaccine (i.m.)	Boost vaccine (i.n.)
1	VSV-Luc	VSV-Luc
2	VSV-H1	VSV-Luc
3	VSV-H1	NS1(1–126)
4	VSV-H1	NS1(1–126)-ΔPAX
5	VSV-Luc	NS1(1–126)-ΔPAX

### Enhanced systemic antibodies following intramuscular prime/intranasal boost vaccination

**Fig 3:**
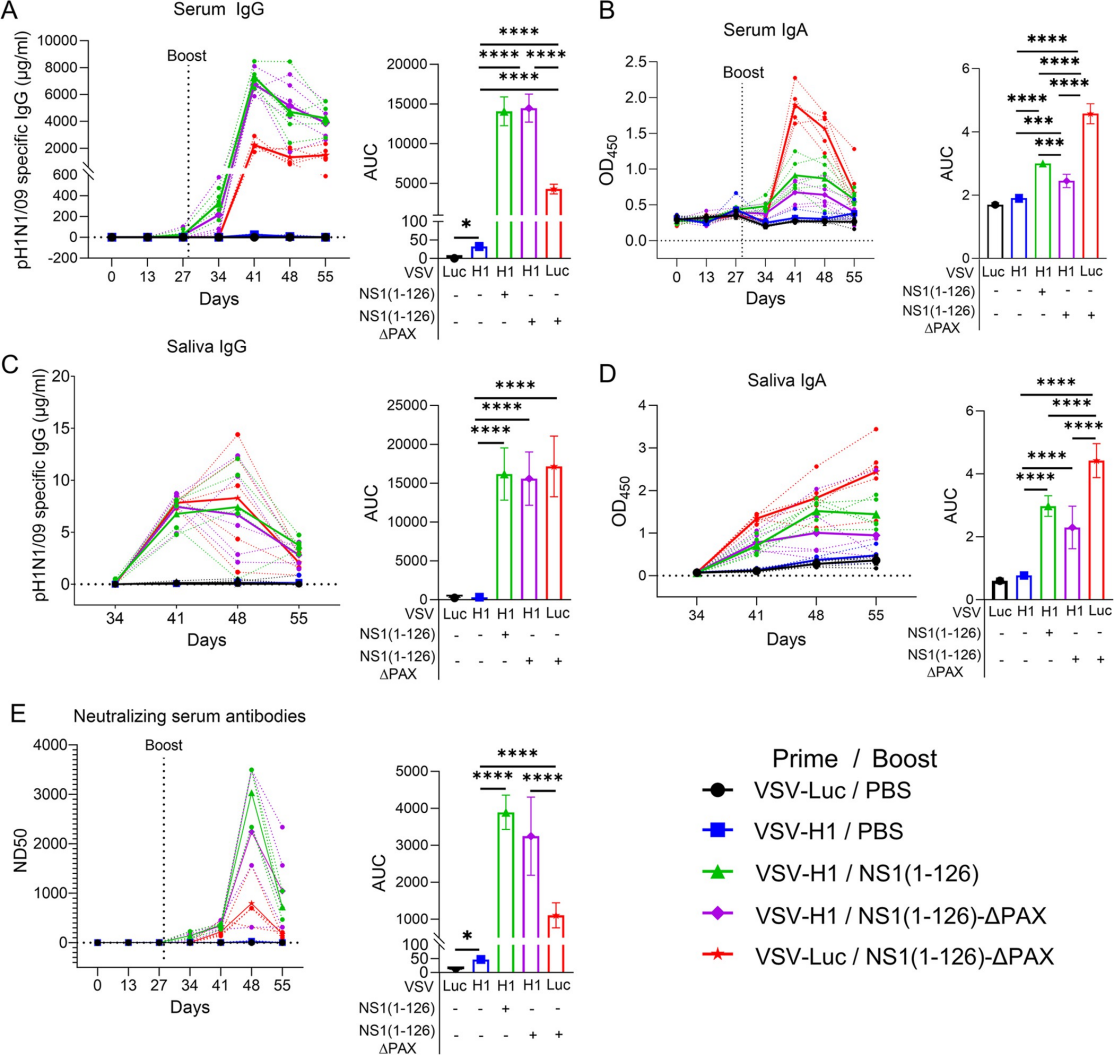
Antibody responses following intramuscular prime/intranasal boost vaccination of pigs. SPF pigs (group size n = 5) were immunized according to the intramuscular prime/intranasal boost vaccination regimen depicted in Fig 2A. (A-D) Analysis of pH1N1/09-specific antibodies by ELISA. The IgG concentrations in serum (A, left panel) and saliva (C, left panel) are depicted by dotted lines for individual animals at each time point analyzed. The bold lines represent the mean values for each animal group for each time point analyzed. The specific IgA levels in serum (B, left panel) and saliva (D, left panel) are represented by dotted lines for the individual animals at each time point analyzed. The bold lines represent the mean values per animal group for each time point. Area under curve (AUC) analysis of the respective data (A-D, right panels). (E) Determination of the virus-neutralizing dose 50% (ND_50_) in the serum of immunized animals. The dotted lines represent the pH1N1/09-neutralizing antibody titres that were detected in the serum of individual animals at the indicated time points. The bold lines represent the mean values for each animal group (E, left panel). AUC analysis of the data (E, right panel). Significant differences were determined using the one-way ANOVA test (*p<0.05, **p<0.01, ***p<0.001, ****p<0.0001).

To assess the immune response following vaccination, serum and saliva were collected at different time points after primary and secondary immunization and analyzed by indirect ELISA. After primary immunization with VSV-H1, virus-specific serum IgG and IgA were not detected (Fig 3A and 3B), but were strongly induced following intranasal boost with the NS1(1–126)-ΔPAX or the NS1(1–126) LAIV. In contrast, significantly lower levels of specific serum IgG were detected if the animals were first immunized with the control vector VSV-Luc and subsequently intranasally boosted with the NS1(1–126)-ΔPAX LAIV (Fig 3A, right panel). With respect to serum IgA, the VSV-Luc/NS1(1–126)-ΔPAX vaccination protocol resulted in higher virus-specific IgA levels compared to the VSV-H1/NS1(1–126) and VSV-H1/NS1(1–126)-ΔPAX vaccination protocols (Fig 3B). Interestingly, the serum IgA response was relatively short lasting with a peak at two weeks followed by a sharp decline (Fig 3B). In contrast, serum IgG levels reached a plateau two weeks after the boost and thereafter decreased only slowly (Fig 3A).

In addition to serum antibodies, we also analyzed the mucosal immune response by measuring the pH1N1/09-specific IgG and IgA antibody levels in saliva by indirect ELISA. Intramuscular immunization with VSV-H1 followed by mock-vaccination with PBS via the nasal route did not result in the induction of detectable levels of IgG in saliva (Fig 3C). In contrast, when animals were primed with VSV-H1 and then boosted with either NS1(1–126) or NS1(1–126)-ΔPAX LAIV, a strong increase in virus-specific secreted IgG was observed between days 34 and 41 post primary vaccination, with no significant differences between the different LAIVs. These antibody levels stayed high until day 48 and then dropped (Fig 3C). With respect to virus-specific IgA in saliva, a small increase in ELISA reactivity was observed for the animals which were primed with either VSV-Luc or VSV-H1 and boosted with PBS (Fig 3D). Considering that this reaction was also found in the absence of H1 expression, it can be considered as a non-specific reaction. In contrast, the animals primed with VSV-H1 and subsequently boosted with either NS1(1–126) or NS1(1–126)-ΔPAX LAIV showed significantly increased IgA responses with the highest IgA levels induced by the VSV-Luc/NS1(1–126)-ΔPAX vaccination regimen (Fig 3D).

Next, we analyzed the induction of virus-neutralizing antibodies. Priming with VSV-H1 resulted in only very low levels of neutralizing antibodies (Fig 3E). However, when the H1-primed animals were boosted via the nasal route with either the NS1(1–126)-ΔPAX or the NS1(1–126) LAIV, a significant increase in virus-neutralizing serum antibodies was detected (Fig 3E, right panel). The ND_50_ values reached a peak of approximately 3000 at day 48 and dropped to an ND_50_ value of approximately 1000 at day 55 post primary immunization (Fig 3E, left panel). In contrast, pigs that had been immunized (i.m.) with the control vector VSV-Luc and subsequently boosted with NS1(1–126)-ΔPAX showed significantly lower neutralizing antibody levels compared to VSV-H1- primed and NS1(1–126)-ΔPAX-boosted animals (Fig 3E, right panel).

### Enhanced CD4^+^ T-cell memory following intramuscular prime/intranasal boost vaccination

**Fig 4:**
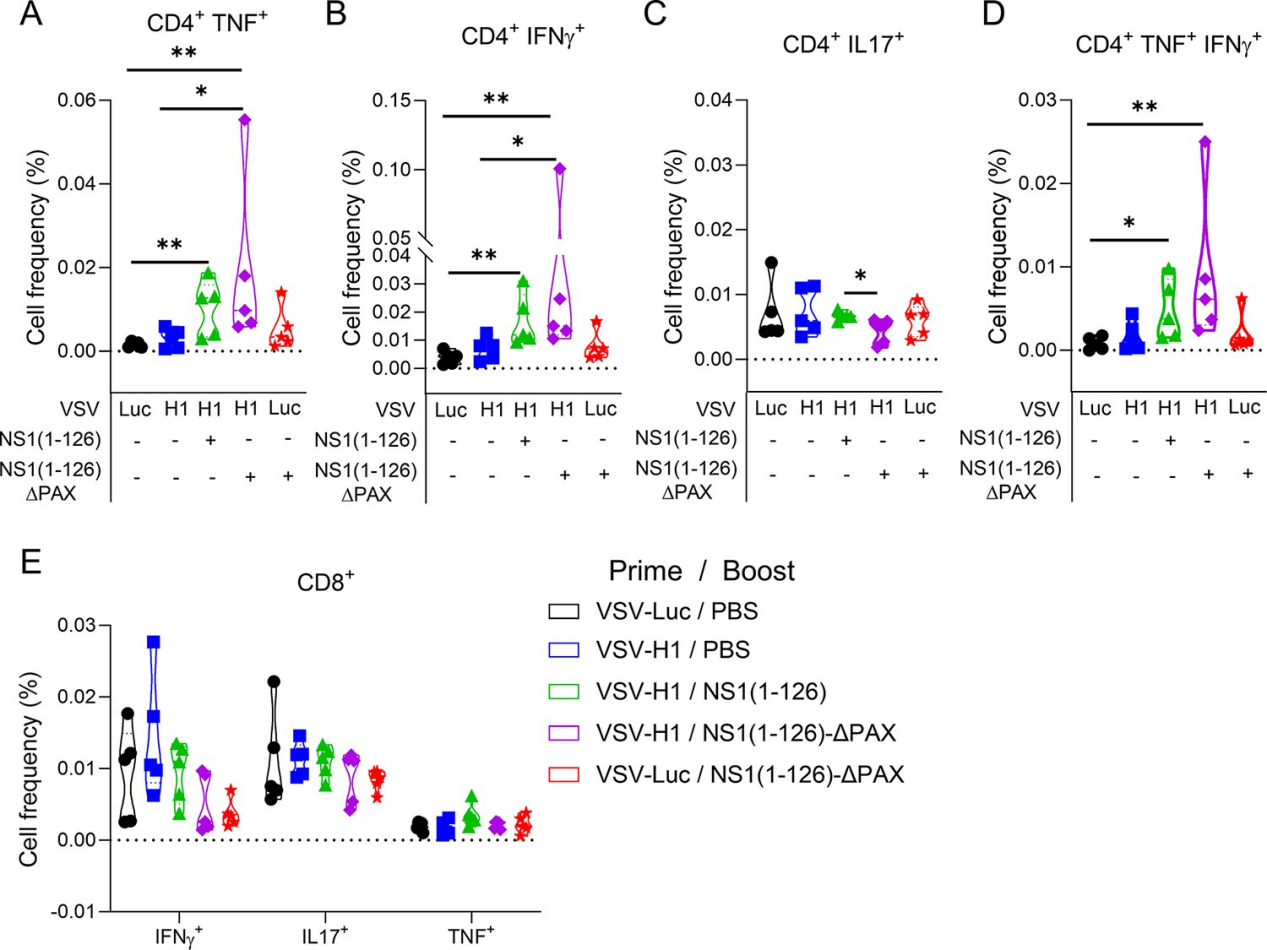
Induction of memory T cell responses in the peripheral blood of pigs following intramuscular prime/intranasal boost vaccination. PBMCs were isolated from immunized pigs at day 48 and restimulated with live pH1N1/09 (see scheme in Fig 2A). (A-D) Intracellular cytokine staining of CD4^+^ T cells for TNF (A), IFNγ (B), IL-17 (C), and TNF/IFNγ double-positive cells (D). The frequency of cytokine-positive cells relative to the total number of CD4^+^ T cells is shown. (E) Frequency of reactivated CD8^+^ cells that were positive for the indicated cytokines. Significant differences were determined using the Mann-Whitney test (*p<0.05, **p<0.01, ***p<0.001, ****p<0.0001).

The cellular arm of the immune system is essential to help in the activation of B cells through the production of cytokines and effector molecules (CD4^+^ T cells), and to clear virus-infected cells (CD8^+^ T cells) [56]. To assess the level of CD4^+^ and CD8^+^ T-cell memory induction by the prime/boost vaccination strategy, PBMCs were collected at day 48 (Fig 2A), restimulated with viral antigen and analyzed by flow cytometry (FCM) for intracellular expression of IFNγ, TNF and IL-17 in CD4^+^ or CD8^+^ T cells (Fig 4). Pigs vaccinated only once with VSV-H1 showed no significant activation of CD4^+^ and CD8^+^ T cells when compared to the control vector group (VSV-Luc). Animals that were primed with VSV-H1 and boosted with either NS1(1–126) or NS1(1–126)-ΔPAX LAIV showed significantly increased numbers of CD4^+^ T cells expressing TNF, IFNγ and both TNF and IFNγ but not IL-17 (Fig 4A–4D). Importantly, this was not observed in the animal group primed with the control vector VSV-Luc and boosted with NS1(1–126)-ΔPAX LAIV (Fig 4A, 4B and 4D; red graphs), demonstrating improved priming of CD4 Th1 cells by the intramuscular prime/intranasal boost protocol. No recall responses were observed for any of the vaccine groups in the CD8^+^ T cell subset (Fig 4E).

### Induction of sterilizing immunity

**Fig 5:**
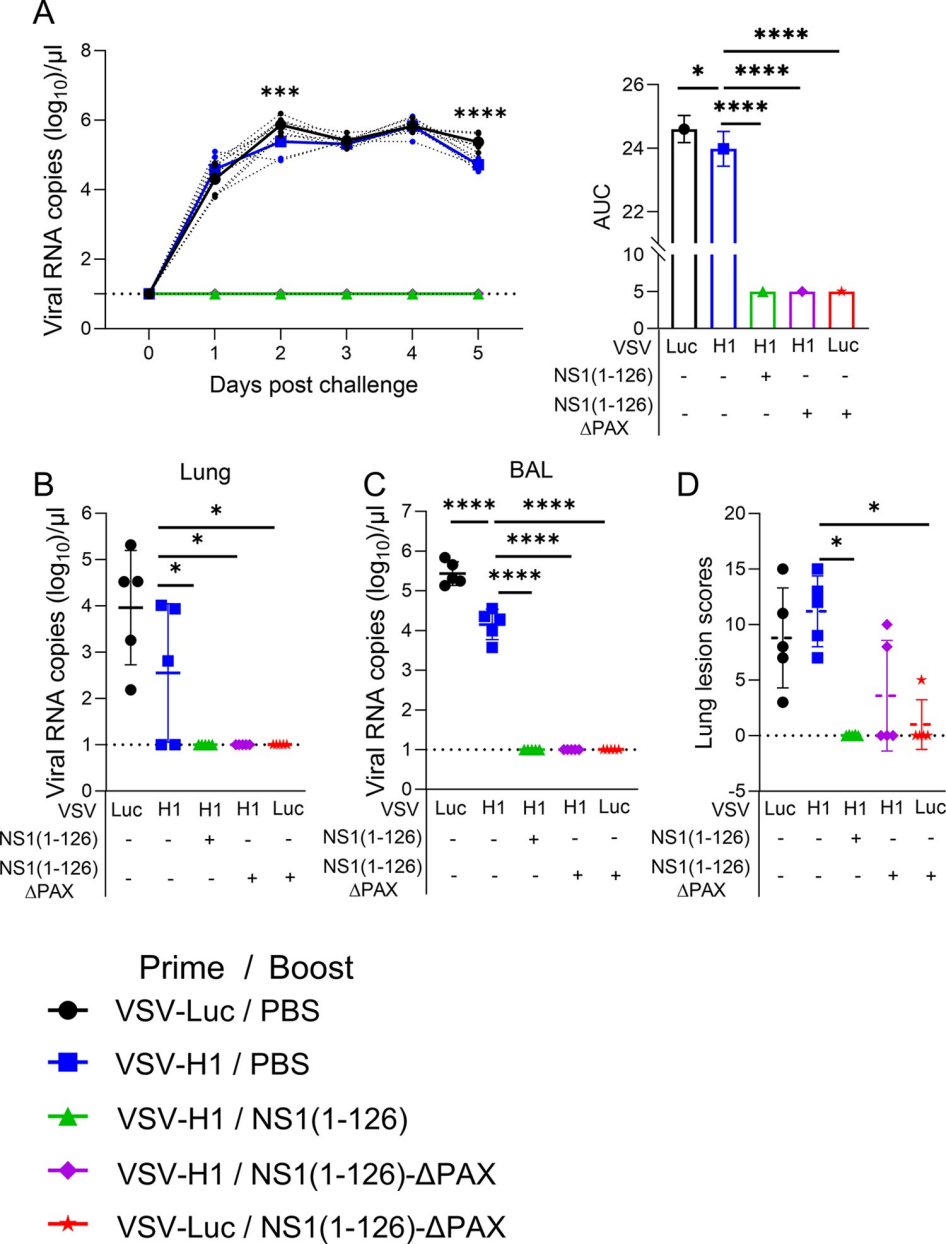
The prime/boost vaccination strategy induces sterilizing immunity to homologous virus challenge. Pigs were vaccinated according to the indicated vaccine combinations and challenged at day 55 with pH1N1/09. **(**A**)** Determination of viral RNA loads in nasal swab samples collected at days 1 to 5 post infection (left panel). The dotted lines represent the RNA loads in nasal swabs collected from individual animals. The bold lines represent the mean values. The AUC analysis of these data is shown in the right panel. (B, C) Viral RNA loads in lung tissue (B) and BAL fluid (C) collected at day 5 post infection. (D) Histopathological lung lesion scores. Significant differences were determined using the one-way ANOVA test (A-C) and the Kruskal-Wallis test (D) (*p<0.05, **p<0.01, ***p<0.001, ****p<0.0001).

To assess whether the intramuscular prime/intranasal boost vaccination would provide protection against infection with the homologous IAV strain, pigs were intranasally inoculated with pH1N1/09 using a dose of 10^6^ TCID_50_ per animal. Regardless of the treatments, none of the infected animals developed clinical signs of disease such as fever (S3 Fig). In swab samples taken from the vaccine groups VSV-H1/NS1(1–126)-ΔPAX, VSV-H1/NS1(1–126), and VSV-Luc/NS1(1–126)-ΔPAX, no viral RNA was detected (Fig 5A and S3 Table), indicating the absence of any virus replication in the respiratory tract of the immunized animals. In contrast, in the VSV-Luc/PBS and VSV-H1/PBS groups, high level virus shedding was observed which reached 10^6^ genome equivalents/μl at day two post infection and stayed at this level until euthanasia at day five (Fig 5A and S3 Table). When the VSV-H1/PBS group was compared to the VSV-Luc/PBS group, a minor but significant reduction of viral RNA was found at days two and five post infection (Fig 5A, right panel).

Following euthanasia of the pigs at day 5 post infection, viral RNA was also measured in lung homogenates. The highest levels were detected in animals of the VSV-Luc/PBS control group, while the VSV-H1/PBS group displayed lower levels of viral RNA (Fig 5B). However, this difference was not significant due to high sample variability. Importantly, as observed in the swab samples, no viral RNA was detected in the lungs of animals that had been immunized with either of the two LAIVs (Fig 5B). With respect to the presence of viral RNA in BAL fluid, the animals of the VSV-H1/PBS group showed a significantly reduced level of viral RNA compared to the VSV-Luc/PBS group (Fig 5C). In contrast, viral RNA was completely absent from the BAL fluid of animals that had been immunized with one of the LAIV candidates (Fig 5C). In agreement with the lack of clinical signs, the histopathological examination of the lungs of the VSV-Luc/PBS control group identified only minor lesions that were typical of interstitial pneumonia. The lung lesion score of the VSV-H1/PBS group was similar high as that of the control group (Fig 5D). In contrast, no lung lesions at all were observed in the VSV-H1/NS1(1–126) group. The lung lesion score of the VSV-Luc/NS1(1–126)-ΔPAX vaccinated animals was also significantly reduced compared to the VSV-H1/PBS group. The VSV-H1/NS1(1–126)-ΔPAX group showed a reduced lung lesion score which however could not be tested as significantly different due to high sample variability.

**Fig 6:**
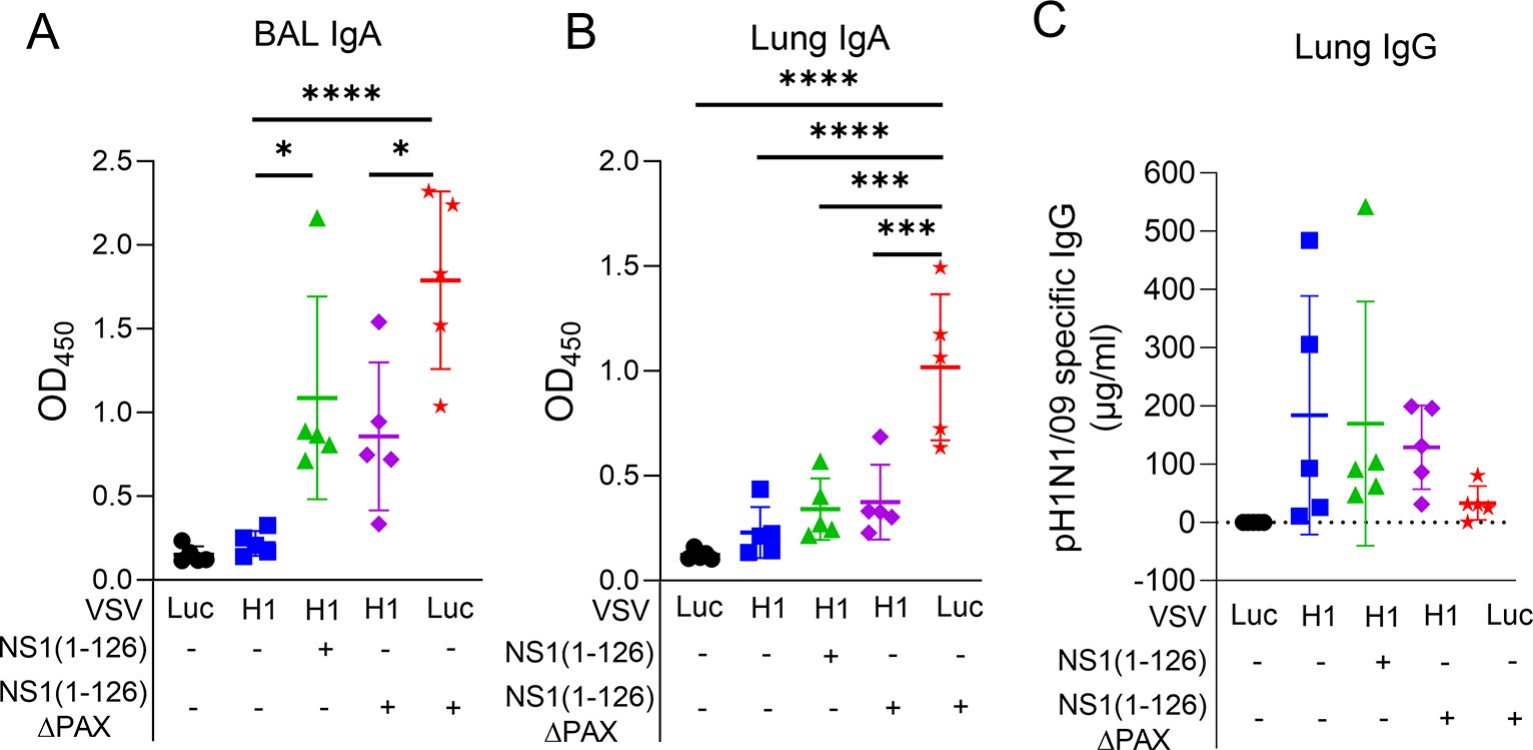
Antibody responses of pigs after nasal challenge infection with pH1N1/09. (A, B) Detection of pH1N1/09-specific IgA in BAL fluid (A) and lung tissue (B) collected 5 days post infection. (C) Detection of pH1N1/09-specific IgG in lung tissue. Significant differences were determined using the one-way ANOVA test (*p<0.05, **p<0.01, ***p<0.001, ****p<0.0001).

The analysis of the mucosal antibody titers in BAL fluid revealed that pigs that had been first primed with VSV-H1 and then boosted with NS1(1–126)-ΔPAX LAIV, had significantly higher IgA levels compared to the VSV-H1/PBS group (Fig 6A). In lung homogenates, only the VSV-Luc/NS1(1–126)-ΔPAX vaccinated animals showed significantly higher IgA levels when compared to the VSV-Luc/PBS and VSV-H1/PBS vaccine groups (Fig 6B). With respect to lung IgG levels, no significant enhancement was found by any of the prime/boost vaccine immunizations even though animals that had been primed with VSV-H1 and boosted with either NS1(1–126) or NS1(1–126)-ΔPAX showed increased pH1N1/09-specific IgG levels by trend (Fig 6C).

Taken together, these results demonstrate that our novel intramuscular prime/intranasal boost vaccination protocol induces enhanced systemic IAV-specific immunity in terms of higher IgG, higher neutralizing antibodies titers and enhanced memory Th1 cells. These systemic responses combined with the induction of local mucosal immunity in the respiratory tract provided sterilizing immunity against homologous virus infection. Importantly, the combination of the intramuscular immunization with VSV-H1 and the intranasal boost using the NS1(1–126)-ΔPAX LAIV enhanced the safety by strongly reducing LAIV shedding and completely preventing shedding of challenge virus.

## Discussion

Recombinant LAIV encoding truncated NS1 proteins have been shown to induce robust adaptive immune responses in human and animal hosts, resulting in high levels of protection against virulent field viruses [40]. For example, LAIV based on A/swine/Texas/4199-2/98 (H3N2) (sTX98) encoding a truncated NS1(1–126) protein protected pigs against infection with homologous and antigen-drifted viruses [47,48,57] or even heterosubtypic H1N2 virus [49,50]. However, sTX98-NS1(1–126) formed reassortants with endemic field strains circulating in the USA at the time, suggesting a significant level of replication of this LAIV candidate in the porcine respiratory tract [50,52]. Consistent with this observation, Vandoorn et al. reported that sTX98-NS1(1–126) was shed from the upper respiratory tract of pigs for several days and caused significant lung pathology [53]. Interestingly, although the NS1(1–126) mutant induced higher levels of type 1 IFN in a porcine renal epithelial cell line than the wild-type virus, no elevated IFN levels were detected in the vaccinated pigs [53]. The present study with pH1N1/09 confirmed nasal shedding of a similar NS1(1–126) LAIV candidate by infected pigs for at least 8 days, although it was reduced compared to the shedding of wild-type virus.

To improve the safety profile of the NS1(1–126) LAIV candidate, we pursued the idea of further attenuating the NS1(1–126) mutant by modifying the PA gene to eliminate PA-X protein expression. The PA-X protein, a small accessory protein translated from the PA mRNA by a frameshift mechanism, has been reported to modulate the innate immune response of the host [58–61]. Characterization of the NS1(1–126)-ΔPAX mutant using an established porcine bronchiolar epithelial cell line showed that this mutant virus induced significantly higher IFN levels than either the wild-type virus or the NS1(1–126) mutant, and was severely restricted in its replication *in vitro* [54]. To our surprise, the NS1(1–126)-ΔPAX mutant was shed from the upper respiratory tract of infected pigs over a prolonged period of time (see Fig 2B). A possible explanation for this discrepancy between the *in vitro* and *in vivo* results could be related to the body temperature of the upper respiratory tract, which is significantly lower than the core body temperature. Indeed, recently published data suggest that the innate immune response of virus-infected primary human airway epithelial cells maintained in the air-liquid interface system at 33°C was significantly lower than that of cells maintained at 37°C [62].

To further reduce the shedding of the LAIV candidates, we did not further attenuate the vaccine virus, as we observed a reduced systemic and mucosal antibody response with the NS1(1–126) LAIV compared to the wild-type virus (see Fig 1F and 1G). We rather preferred an alternative vaccination strategy based on a heterologous prime/boost protocol. Our concept combined the LAIV approach with the excellent ability of VSV-based vectors to induce systemic antibody and T cell responses after intramuscular injection [55]. To this end, we used the propagation-defective VSV-H1 replicon particles expressing HA for intramuscular priming and the NS1(1–126) and/or PA-X-modified LAIV for nasal boosting to induce local protection. This approach significantly reduced LAIV shedding. In particular, excretion of the NS1(1–126)-ΔPAX double mutant was significantly reduced in terms of duration and RNA load. However, RT-qPCR analysis of some swab samples still showed CT values in the range of 25 to 30 (S2 Table), indicating that viral shedding was not completely blocked. To optimize our prime/boost strategy in future experiments, the animals could be primed with VSV-H1 together with VSV-vectored NA antigen. As antibodies to the NA antigen have been shown to interfere with virus spread [63,64], this strategy may further reduce LAIV shedding.

Both VSV-H1 and NS1(1–126)-ΔPAX LAIV are capable of driving intracellular expression of IAV antigens and efficiently stimulate both the humoral and the cellular arms of the immune system. However, a single immunization usually elicits only a weak and short-lived immune response. It is only after a second immunization that memory B and T cells are restimulated, leading to their proliferation and the generation of more and long-lived plasma cells that secrete antigen-specific antibodies with increased affinity [65]. Consistent with this view, we found that compared to single immunization with either VSV-H1 or NS1(1–126)/ΔPAX LAIV, intranasal immunization of VSV-H1-primed animals with NS1(1–126)/ΔPAX LAIV significantly boosted the virus-specific serum IgG response (Fig 3A) as well as virus-neutralizing antibody titers (Fig 3E). In addition, single immunization with NS1(1–126)/ΔPAX LAIV did not result in significantly increased memory T cell responses, whereas the prime/boost vaccination with VSV-H1/NS1(1–126) or VSV-H1/NS1(1–126)/ΔPAX did (Fig 4). However, future work is required to determine whether this enhanced memory T cell response would result in prolonged protection against IAV infection.

Previous studies have shown that a single intranasal immunization of pigs with NS1-truncated H3N2 LAIV resulted not only in complete protection against homologous virus challenge, but also in almost complete protection against antigen-drifted virus and partial protection against heterosubtypic IAV [48]. It will be of high interest to analyze in future experiments whether our prime/boost vaccination regimen and the induction of a strong memory T cell response will also provide protection against antigen-drifted IAV. In this regard, the inclusion of the NA antigen in the VSV-based vaccine used for priming may further broaden the antiviral immune response. Although sterilizing immunity to antigen-drifted viruses may not be achieved, we expect that the shedding and transmission of antigen-drifted challenge viruses will be largely reduced compared to immunization with current inactivated vaccines.

Pre-existing immunity to IAV can significantly affect the efficacy of LAIV because HA- and/or NA-specific antibodies directed to the vaccine virus can interfere with its replication and reduce antigen expression [66]. This could be particularly problematic if the LAIV is overly attenuated or the vaccine dose used is too low. Accordingly, our experiments also showed that the intramuscular priming with VSV-H1 reduced LAIV shedding and resulted in lower mucosal antibody levels compared to the LAIV-only strategy. However, the prime/boost vaccination regimen was more immunogenic in terms of induction of systemic IgG and neutralizing antibodies as well as Th1 memory cells and was as efficient as the LAIV-only approach in mediating complete protection from challenge infection, as indicated by the absence of lung lesions and the lack of viral RNA in respiratory tissues. Based on our findings, we hypothesize that pre-existing anti-influenza immunity may even enhance vaccine efficacy, provided that an appropriate LAIV is administered.

In conclusion, the present novel prime/boost vaccination protocol provides a basis for the development of alternative next-generation vaccination strategies that will help to more effectively control seasonal influenza epidemics and persistent circulation of IAV in livestock. In particular, the mucosal immunity induced by this vaccine strategy may reduce the spread of IAV and help to improve the level of herd immunity. As this prime/boost vaccination strategy can be easily and timely adapted to emerging IAV subtypes, it may also be useful in the control of future IAV pandemics.

## Material and methods

### Ethics statement

The study was performed according to Swiss laws (the Animal Welfare Act TSchG SR 455, the Animal Welfare Ordinance TSchV SR 455.1, and the Animal Experimentation Ordinance TVV SR 455.163). All experiments were reviewed by the committee on animal experiments of the canton of Bern and approved by the cantonal veterinary authority under the license number BE64/2020.

### Cells

Madin-Darby canine kidney type II cells (MDCK-II) were kindly provided by Georg Herrler (University of Veterinary Medicine, Hannover, Germany) and maintained with Minimum Essential Medium (MEM, Thermo Fisher Scientific, Basel, Switzerland; cat. no. 31095–029) supplemented with 5% of fetal bovine serum (FBS; Pan Biotech, Aidenbach, Germany; cat. no. P30-3033). Human embryonic kidney (HEK) 293T cells (American Type Culture Collection (ATCC), Manassas, USA; cat. no. CRL-3216) were maintained with Dulbecco’s Modified Eagle’s Medium (DMEM, Thermo Fisher Scientific; cat. no. 32430–027) supplemented with 10% FBS. Baby hamster kidney 21 (BHK-21) fibroblasts were obtained from ATCC (cat. no. CCL-10) and maintained in Glasgow’s Minimal Essential Medium (GMEM, Thermo Fisher Scientific, cat. no. 21710–025) supplemented with 5% FBS. BHK-G43 cells, a transgenic BHK-21 cell clone expressing VSV glycoprotein G in a regulated manner, was maintained in GMEM supplemented with 5% FBS [67]. All cells were grown at 37°C in a 5% CO_2_ atmosphere.

### Generation of recombinant influenza virus

The pHW2000 plasmids encoding the 8 RNA segments of A/Hamburg/4/2009 (H1N1) (GenBank accession nos.: GQ166207, GQ166209, GQ166211, GQ166213, GQ166215, GQ166217, GQ166219, GQ166221) were originally provided by Hans-Dieter Klenk (University of Marburg, Marburg, Germany). The pHW2000 plasmids encoding RNA segments 3 (PA) and 8 (NS1, NEP) were modified as previously described [33]. Briefly, four stop codons were inserted at position 126 of the NS1 open reading frame leading to a truncated protein lacking the last 93 amino acids. This virus is termed “NS1(1–126)”. To eliminate PA-X expression, four nucleotides were changed at position 191 without modifying the amino acid sequence of the PA protein. This virus was termed “NS1(1–126)-ΔPAX”. Recombinant viruses were generated using the eight-plasmid system as previously described [33,44] and passaged two times on MDCK-II cells with FBS-deficient medium containing 1% penicillin/streptomycin and 1 μg/ml of N-tosyl-L-phenylalanine chloromethyl ketone-treated trypsin (TPCK-trypsin, Merck KGgA, Darmstadt, Germany, cat. no. 4370285). Infectious virus titers were determined on MDCK-II cells as previously described [68], using a monoclonal antibody directed to the influenza virus nucleoprotein (clone H16-L10-4R5; ATCC, HB-65) for detection of infected cells by indirect immunofluorescence.

### Generation of recombinant VSV vector vaccine

The HA gene of A/Hamburg/4/2009 (H1N1) (GenBank acc. no. GQ166213) was amplified by PCR using the Phusion DNA polymerase and inserted into the *Mlu*I and *Bst*EII sites of the pVSV*ΔG(HA_H5-HP_) plasmid [46], resulting in the pVSV*ΔG(H1) plasmid. Recombinant vesicular stomatitis virus (VSV) replicon particles were generated as previously described [55,69,70]. Briefly, BHK-G43 cells were first infected with modified vaccinia virus Ankara encoding the T7 phage RNA polymerase (MVA-T7, kindly provided by Gerd Sutter from Ludwig-Maximilians-Universität, München, Germany) using a multiplicity of infection (moi) of 3 focus-forming units (ffu) per cell. Subsequently, the cells were transfected with pVSV*ΔG(H1) along with three plasmids encoding the VSV N, P, and L proteins; all under the control of the T7 promoter. The cells were incubated for 24 h at 37°C and 5% CO_2_ in the presence of 10^−9^ M of mifepristone (Merck KGaA, Darmstadt, Germany) to allow expression of the G protein. Then, the cells were trypsinized and seeded along with fresh BHK-G43 cells into a T75 flask and incubated with GMEM containing 5% FBS and 10^−9^ M mifepristone for 24 h at 37°C and 5% CO_2_. The supernatant was harvested, and cell debris removed by low-speed centrifugation, and the supernatant passed through a 0.2 μm pore-size filter. The recombinant VSV-H1 vector was propagated on mifepristone-treated BHK-G43 cells and stored in aliquots at -70°C. Infectious virus titres were determined on BHK-21 cells. The recombinant VSV-Luc vector has been generated previously and was propagated accordingly [69].

### Animal experiments

For the first animal experiment, 15 healthy 10-weeks old Large White conventional pigs were purchased from Agroscope (competence center of the Swiss confederation in the field of agricultural and agri-food research). The animals were randomly allocated into three groups each containing five pigs of mixed sex. The animals were intranasally immunized with 10^6^ ffu/animal of either pH1N1/09, pH1N1/09-NS1(1–126), or VSV-Luc as control. Body temperature and clinical symptoms of disease were monitored for the following 12 days. Oro-nasal swab samples were taken daily for the first 12 days post immunization to monitor virus shedding. Serum and saliva samples were taken once a week for a total period of six weeks after immunization to monitor for systemic and local antibody responses. Six weeks after immunization, the animals were euthanized by electrical stunning and subsequent exsanguination. Bronchoalveolar lavage (BAL) was prepared immediately after exsanguination.

For the second animal experiment, 25 healthy 10-weeks old specific pathogen-free (SPF) Swiss Large White pigs from the Institute of Virology and Immunology (IVI, Mittelhäusern) breeding facility were used. The animals were tested seronegative for the influenza A virus nucleoprotein by using a commercial ELISA (ID Screen Influenza A Antibody Competition Multi-species ELISA, ID-Vet, Montpellier, France, cat. no, FLUACA). The animals were divided randomly into five groups each containing five pigs of mixed sex and immunized via the intramuscular route using a dose of 10^8^ ffu/pig of either VSV-H1 or the control vector VSV-Luc (**Table 1**). Temperature and clinical symptoms were monitored daily for seven days, and serum samples were prepared at days 13 and 27 after primary immunization (**Fig 2A**). At day 28 after primary immunization, the animals were immunized via the nasal route with LAIV (see **Table 1**) using a dose of 10^5^ ffu/pig.

To this end, an intranasal mucosal atomization device (MAD Nasal, Teleflex Medical Europe Ltd., Ireland, cat. no, MAD300) plugged to a 10-ml syringe was employed. Body temperature and clinical symptoms were monitored for one week following the second (boost) immunization. Swabs samples were taken daily, suspended in 2 ml of MEM, and stored at -70°C prior to use. Serum and saliva samples were taken every week for four weeks and stored at -20°C prior to serological testing. Blood samples were collected at day 49 (three weeks post boost) for isolation of PBMCs and T cell reactivation experiments. At day 56, the animals were infected with wild-type pH1N1/09 via the nasal route using 10^6^ ffu/pig. Body temperature and clinical symptoms were monitored, and serum and saliva samples were collected daily. Five days after challenge infection, the animals were euthanized by electrical stunning and subsequent exsanguination. Immediately after exsanguination, the lung’s right cranial lobe was prepared for viral RNA detection and lung samples were fixed with formalin as indicated below for histological analysis. Bronchoalveolar lavage was prepared for detection of virus-specific antibodies and viral RNA.

### ELISA

For detection of antibodies directed against pH1N1/09, 96-well plates (Nunc MaxiSorp) were coated overnight at room temperature with 1 μg/well of heat-inactivated A/H1N1/09 (56°C, 30 min). Plates were blocked for 1h at 37°C with 200 μl/well of 1% bovine serum albumin (BSA) in PBS containing 0.05% (v/v) Tween 20. Serum, saliva, and BAL samples were added in duplicates at the right dilution and incubated for 1 h at 37°C. Ten-fold serial dilutions of pH1N1/09 HA-specific antibody (Thermo Fisher, cat. no. PA5-81645) were used as standard. Plates were washed with PBS (without Ca^2+^ and Mg^2+^) containing 0.05% (v/v) of Tween 20 and incubated with horse radish peroxidase (HRP)-conjugated anti-swine IgG (Abcam, cat. no. ab6915) or anti-swine IgA (Bethyl, cat. no. A100-102P) diluted 1/2000 and 1/50’000, respectively. The cells were washed three times with 200 μl/well of PBS/Tween and incubated for 10 min at room temperature with 50 μl/well of 3,3`,5`,5`-tetramethylbenzidine (TMB)/H_2_O_2_ peroxidase substrate (Merck KGaA, cat. no. T4444). The reaction was stopped by addition of 50 μl/well of 1 M HCl and absorbance read at 450 nm using a GloMax microplate reader (Promega, Dübendorf, Switzerland).

### Virus neutralization test

Porcine sera were serially diluted in MEM medium (two-fold or five-fold dilution steps) and added in quadruplicates to 96-well microtiter plates (50 μl/well). To each well 50 μl of pH1N1/09 (2000 ffu/ml) were added and incubated for 1 h at 37°C. The antibody/virus mix was then added to MDCK-II cells that were grown to confluence in 96-well cell culture plates and incubated for 1 h at 37°C and 5% CO_2_. The cells were washed once and incubated with fresh medium (100 μl/well) for 24 h at 37°C and 5% CO_2_. The cells were fixed with 4% formaldehyde solution and immuno-stained as previously described [45]. The 50% neutralizing dose (ND_50_) was calculated using the Spearman-Kärber method [71].

### RNA extraction and RT-qPCR

All samples were directly transferred to 700 μl of RA1 lysis buffer (Macherey-Nagel, Düren, Germany, cat. no. 740961) containing 1% β-mercaptoethanol. Organs were homogenized in RA1 lysis buffer using a tissue bullet blender (Next Advanced Inc., Troy, NY, USA). Total RNA was extracted from the lysates using the NucleoMag Vet kit (Macherey-Nagel, cat.no. 744200) according to the manufacturer’s protocol. Reverse transcription from RNA to cDNA and real-time quantitative PCR (qPCR) were performed with the QuantStudio 5 real-time PCR system (Thermo Fisher Scientific) using the AgPath-ID One-Step RT-PCR kit (Life Technologies, Zug, Switzerland, cat. no. AM1005) and vRNA segment 7-specific oligonucleotide primers and probe [72,73]. Data were acquired and analyzed using the Design and Analysis Software v1.5.2 (Thermo Fisher Scientific). Quantification was performed using an internal standard based on the IAV RNA segment 7.

### T cell assay

Peripheral blood mononuclear cells (PBMC) were isolated from 50 ml of blood in EDTA per pig. For each animal, five million PBMCs were seeded per well (12-well plate format) in triplicates and stimulated for 14 h at 39°C and 5% CO_2_ with pH1N1/09 (moi of 0.1 ffu/cell). Thereafter, brefeldin A at 3 μg/ml (Invitrogen, cat. no. 00-4506-51) was added to the cells and incubated for another 4 h. The cells were washed twice and stained with the fixable Aqua Dead Cell Stain kit (Thermo Fisher, cat. no. L34957). The CD4^+^ and CD8^+^ T lymphocyte cells were labeled by incubation with anti-CD4 IgG2b (clone 74-12-4, hybridoma kindly obtained from Dr. Joan Lunney, USDA Beltsville, MD, USA [74]) and anti-CD8β IgG2a (PG164A, BioTechne, Basel, cat. no. NBP2-60955), followed by isotype-specific AlexaFluor-488 (Thermo Fisher, cat. no. A21141) and PE-Cy7 conjugates (Abcam, cat.no. ab130787). After fixation and permeabilization, anti-IFNγ-PE diluted 1/200 (P2G10, BD Biosciences, cat. no. 561481), anti-TNF-AF647 diluted 1/20 (Mab11, Biolegend, cat. no. 502916) and anti-IL-17 diluted 1/5 (SCPL1362, BD Bioscience, cat. no. 560436) were added. Data were acquired on FACS Canto-II (BD Bioscience) and analyzed using FlowJo software, version 10 (BD Bioscience).

### Histopathology

Three lung samples of approximately 1 cm in diameter from the same location of the right lung: apical lobe, medial lobe, and caudo-dorsal area of the diaphragmatic lobe, were systematically taken and placed in 4% formalin. In addition, when macroscopic lesions compatible with broncho-interstitial pneumonia were observed, up to three additional samples from the affected lobes were taken for histological examination. The samples were trimmed and placed in two different cassettes per pig separately, with one cassette containing the three systematically collected lung sections and the other containing up to three lung sections with macroscopic lesions. The microscopic evaluation was made based on the previously reported Morgan score [75,76]. Necrosis of the bronchiolar epithelium, airway inflammation, perivascular/bronchiolar cuffing, alveolar exudates, and septal inflammation, were scored from 0–4 points each (0: none, 1: minimal, 2: mild, 3: moderate, and 4: severe). All the parameters were added to obtain the scoring per slide (0–20 points), per animal (sum of slide 1 and 2, 0–40 points) and per group (mean score per animal for each group, 0–40 points).

### Statistical analysis

Data analysis and figures were done using GraphPad Prism 8 Software (GraphPad Software). One-way and two-way ANOVA tests with multiple comparisons, Kruskal-Wallis test, and Mann-Whitney tests were used to determine statistical significance in experimental data (detection of viral RNA by RT-qPCR, antibody titers, and T cell recall responses). P values lower than 0.05 were considered as statistically significant.

## Supporting information

S1 Fig Recording of body temperature following immunization of pigs with pH1N1/09, NS1(1–126) LAIV, or VSV-Luc. Rectal body temperature was recorded at the indicated days post immunization. Mean values and standard deviations are shown for each animal group (n = 5).
(TIF)

S2 Fig Recording of body temperature intramuscular prime/intranasal boost vaccination of pigs. (A) Rectal body temperatures of pigs at the first eight days following primary immunization (i.m.). (B) Rectal body temperature of pigs at the first 10 days after the intranasal immunization with the indicated LAIV. Mean values and standard deviations are shown for each animal group (n = 5).
(TIF)

S3 Fig Recording of body temperature in immunized pigs following challenge infection with pH1N1/09. Pigs were immunized according to the indicated prime/boost vaccination protocol and subsequently challenged with pH1N1/09 via the intranasal route. The rectal body temperature of the animals was recorded from one day prior to challenge to 3 days post challenge. Mean values and standard deviations are shown for each animal group (n = 5).
(TIF)

S1 Table Viral RNA loads of nasal swab samples collected following intranasal infection of pigs with either pH1N1/09 or NS1(1–126) LAIV. RNA was extracted from nasal swab samples collected at the indicated days and analyzed for the presence of genomic RNA segment 7 by RT-qPCR. Mean Ct values of duplicate RT-qPCR experiments are shown.
(TIF)

S2 Table Viral RNA loads of nasal swab samples collected from pigs following intramuscular prime/intranasal boost immunization. RNA was extracted from nasal swab samples collected at the indicated days post intranasal boost immunisation with LAIV. The extracted RNA was analyzed for the presence of genomic RNA segment 7 by RT-qPCR. Mean Ct values of duplicate RT-qPCR experiments are shown.
(TIF)

S3 Table Viral RNA loads of nasal swab samples collected after challenge infection of immunized pigs. RNA was extracted from nasal swab samples collected at the indicated days post infection of pigs with pH1N1/09. The extracted RNA was analyzed for the presence of genomic RNA segment 7 by RT-qPCR. Mean Ct values of duplicate RT-qPCR experiments are shown.
(TIF)
