# Supplementary figures and images for: Evaluation of a novel intramuscular prime/intranasal boost vaccination strategy against influenza in the pig model
Source: PLoS Pathog. 2024 Aug 8;20(8):e1012393. doi: 10.1371/journal.ppat.1012393 (PMC11309389; doi:10.1371/journal.ppat.1012393)

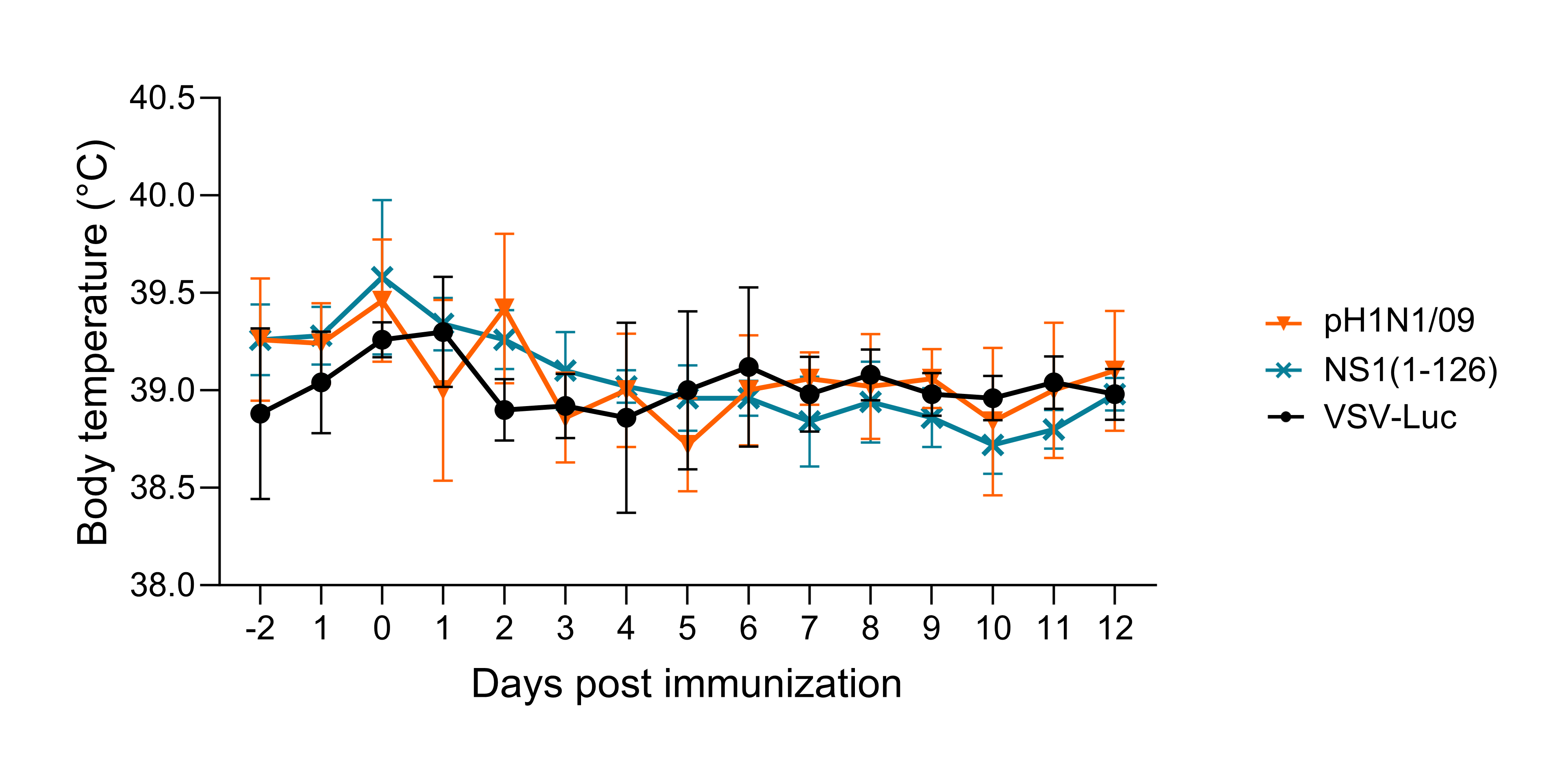

Supplement: S1 Fig — Rectal body temperature was recorded at the indicated days post immunization. Mean values and standard deviations are shown for each animal group (n = 5). (TIF) [file ppat.1012393.s001.tif]

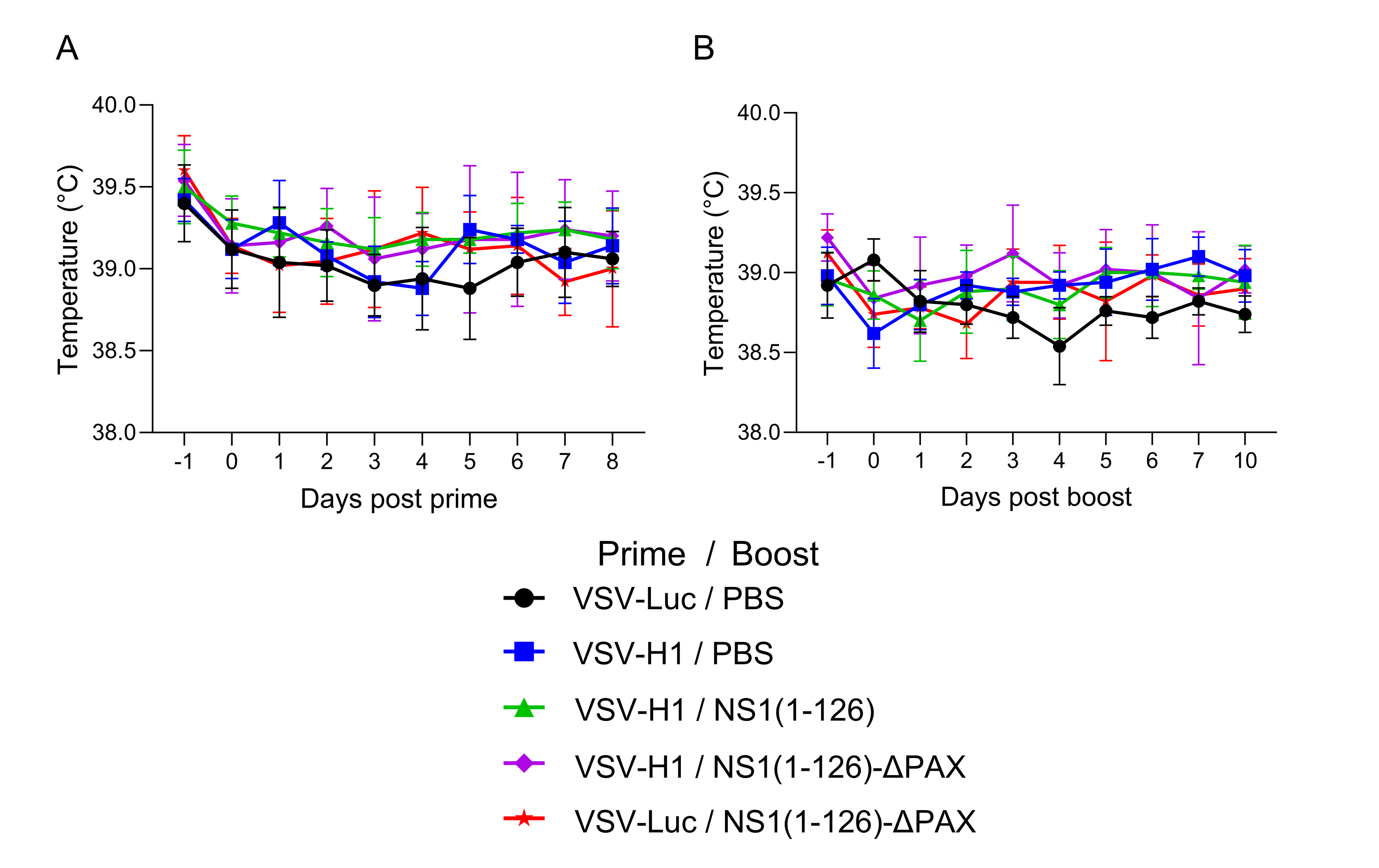

Supplement: S2 Fig — (A) Rectal body temperatures of pigs at the first eight days following primary immunization (i.m.). (B) Rectal body temperature of pigs at the first 10 days after the intranasal immunization with the indicated LAIV. Mean values and standard deviations are shown for each animal group (n = 5). (TIF) [file ppat.1012393.s002.tif]

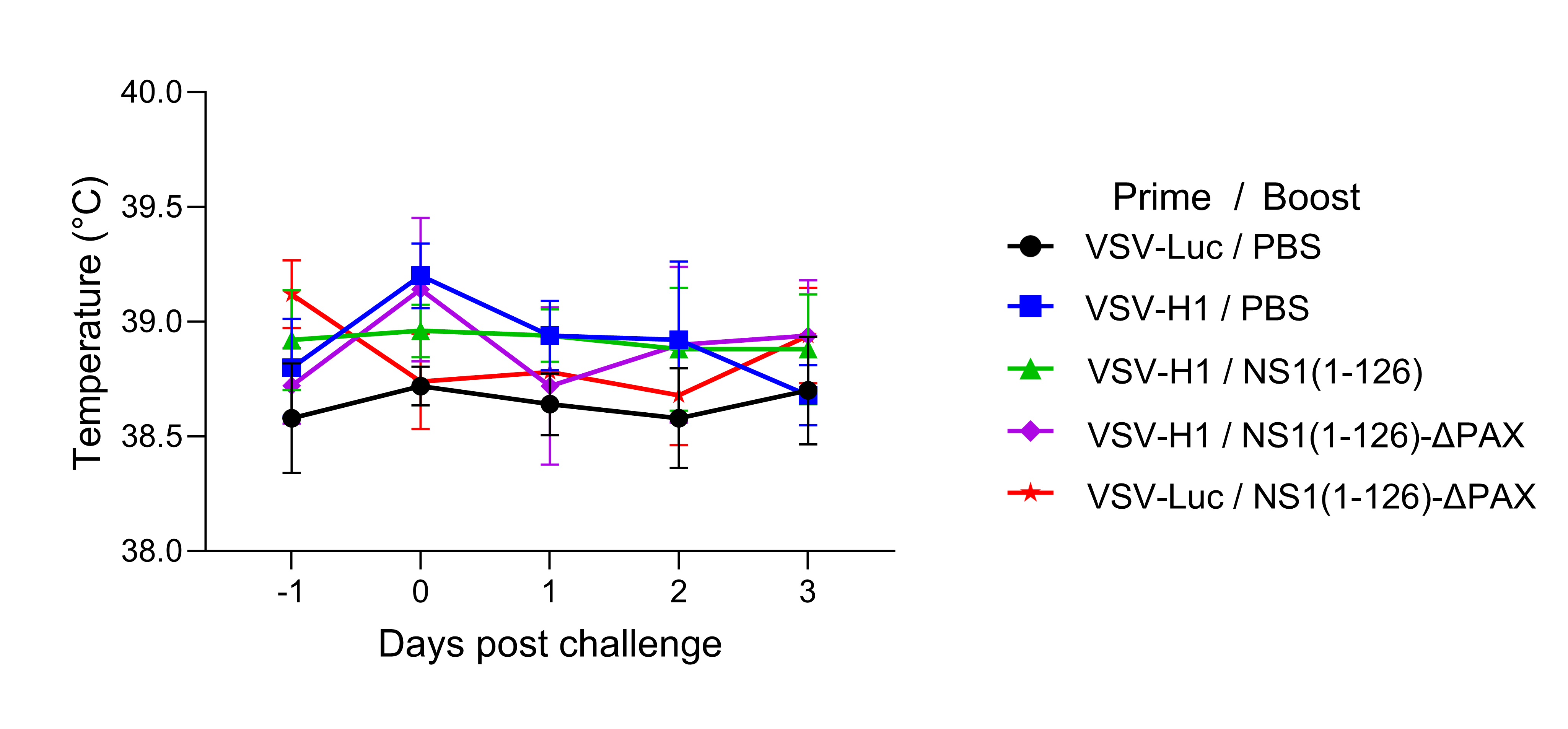

Supplement: S3 Fig — Pigs were immunized according to the indicated prime/boost vaccination protocol and subsequently challenged with pH1N1/09 via the intranasal route. The rectal body temperature of the animals was recorded from one day prior to challenge to 3 days post challenge. Mean values and standard deviations are shown for each animal group (n = 5). (TIF) [file ppat.1012393.s003.tif]

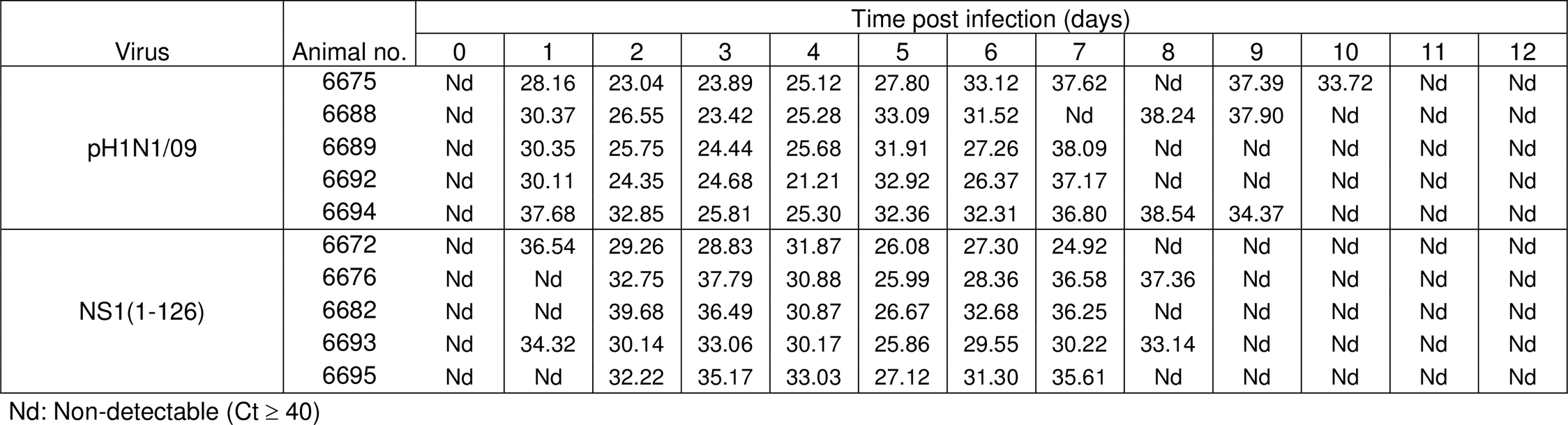

Supplement: S1 Table — RNA was extracted from nasal swab samples collected at the indicated days and analyzed for the presence of genomic RNA segment 7 by RT-qPCR. Mean Ct values of duplicate RT-qPCR experiments are shown. (TIF) [file ppat.1012393.s004.tif]

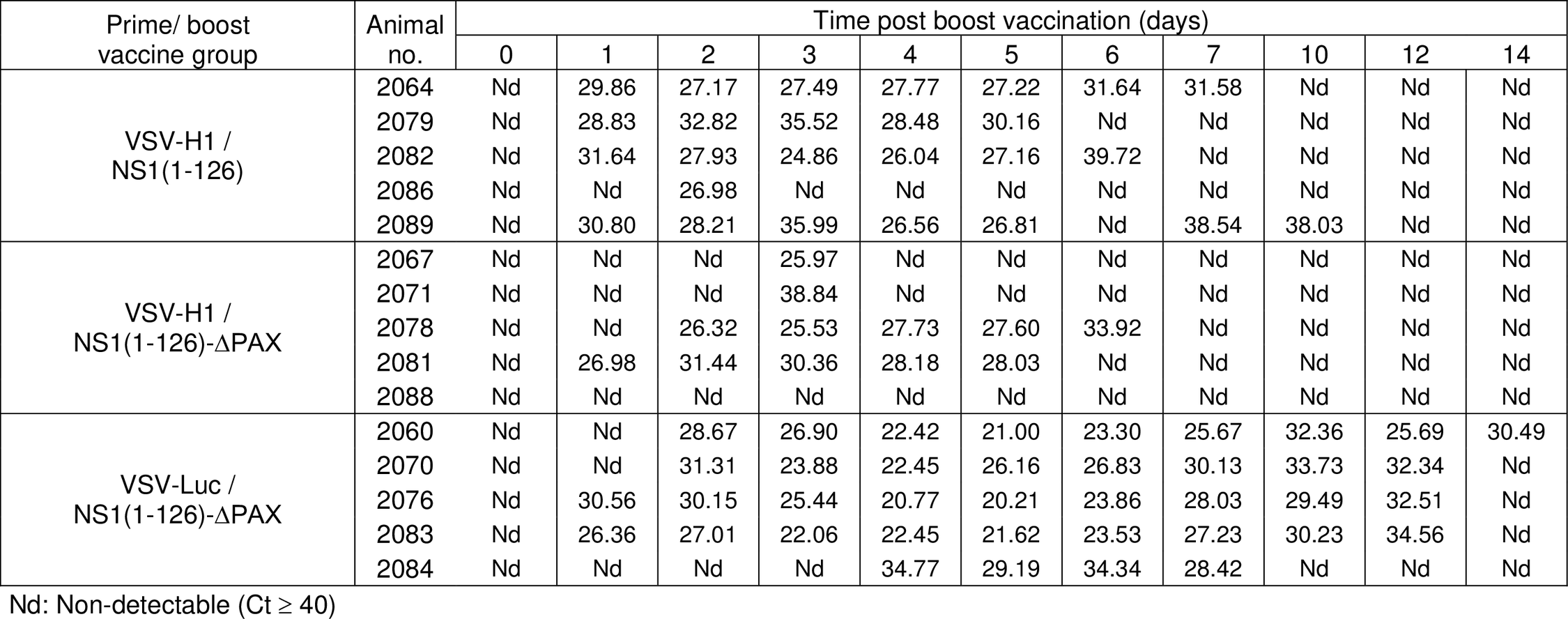

Supplement: S2 Table — RNA was extracted from nasal swab samples collected at the indicated days post intranasal boost immunisation with LAIV. The extracted RNA was analyzed for the presence of genomic RNA segment 7 by RT-qPCR. Mean Ct values of duplicate RT-qPCR experiments are shown. (TIF) [file ppat.1012393.s005.tif]

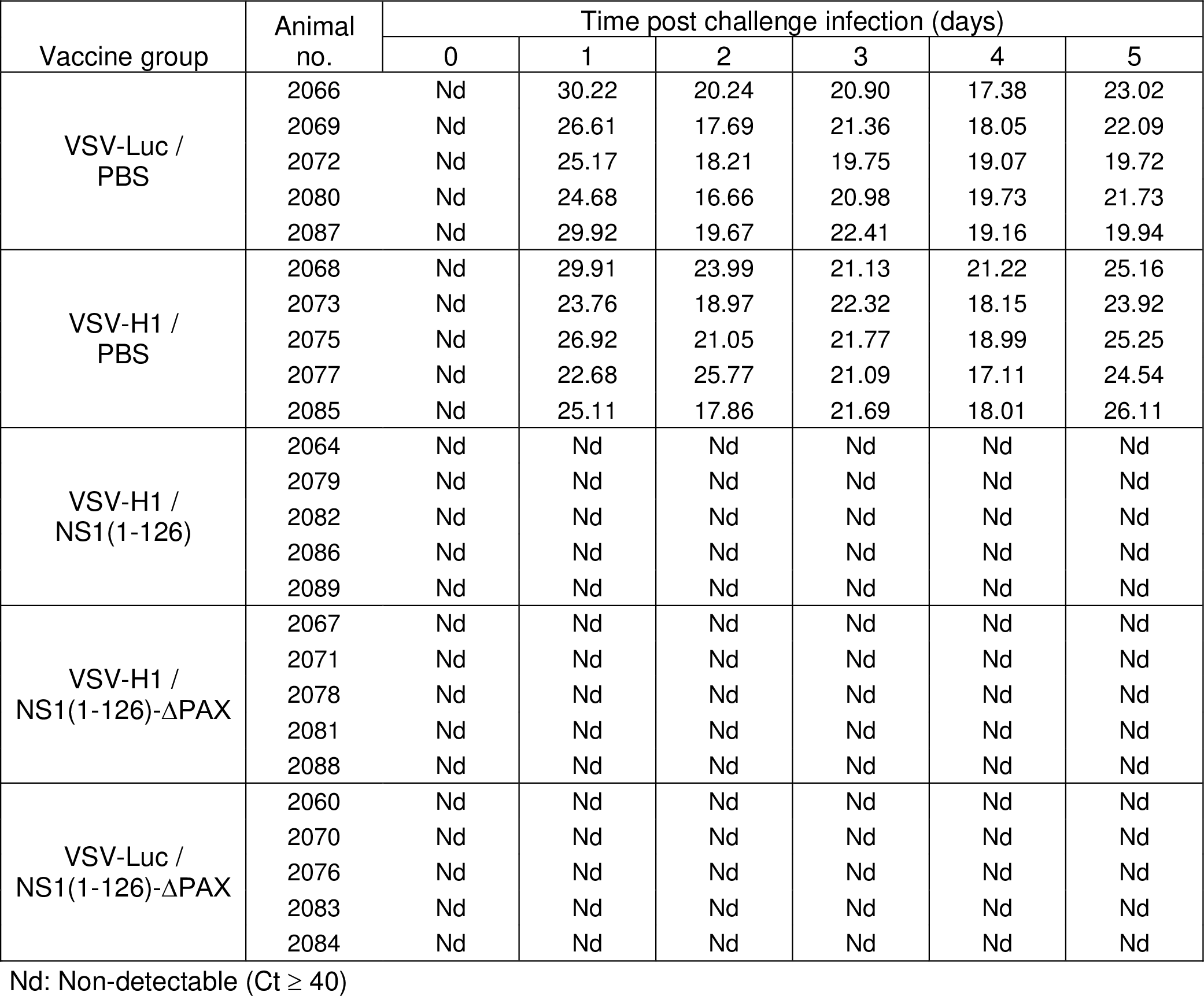

Supplement: S3 Table — RNA was extracted from nasal swab samples collected at the indicated days post infection of pigs with pH1N1/09. The extracted RNA was analyzed for the presence of genomic RNA segment 7 by RT-qPCR. Mean Ct values of duplicate RT-qPCR experiments are shown. (TIF) [file ppat.1012393.s006.tif]
